# Stability of motor-nonmotor subtype in early-stage Parkinson’s disease

**DOI:** 10.3389/fnagi.2022.1040405

**Published:** 2022-11-10

**Authors:** Yi Xiao, Qianqian Wei, Ruwei Ou, Yanbing Hou, Lingyu Zhang, Kuncheng Liu, Junyu Lin, Tianmi Yang, Qirui Jiang, Huifang Shang

**Affiliations:** ^1^Laboratory of Neurodegenerative Disorders, Department of Neurology, Rare Disease Center, National Clinical Research Center for Geriatric, West China Hospital, Sichuan University, Chengdu, Sichuan, China; ^2^Health Management Center, West China Hospital of Sichuan University, Chengdu, Sichuan, China

**Keywords:** follow-up studies (MeSH), Parkinson’s disease, prognosis, subtype, stability

## Abstract

**Background:**

The different clinical characteristics and prognostic values of the motor-nonmotor subtypes of Parkinson’s disease (PD) have been established by previous studies. However, the consistency of motor-nonmotor subtypes in patients with early-stage Parkinson’s disease required further investigation. The present study aimed to evaluate the consistency of motor-nonmotor subtypes across five years of follow-up in a longitudinal cohort.

**Materials and methods:**

Patients were classified into different subtypes (mild-motor–predominant, intermediate, diffuse malignant; or tremor-dominant, indeterminate, postural instability and gait difficulty) according to previously verified motor-nonmotor and motor subtyping methods at baseline and at every year of follow-up. The agreement between subtypes was examined using Cohen’s kappa and total agreement. The determinants of having the diffuse malignant subtype as of the fifth-year visit were explored using logistic regression.

**Results:**

A total of 421 patients were included. There was a fair degree of agreement between the baseline motor-nonmotor subtype and the subtype recorded at the one-year follow-up visit (κ = 0.30 ± 0.09; total agreement, 60.6%) and at following years’ visits. The motor-nonmotor subtype had a lower agreement between baseline and follow-up than did the motor subtype. The baseline motor-nonmotor subtype was the determinant of diffuse malignant subtype at the fifth-year visit.

**Conclusion:**

Many patients experienced a change in their motor-nonmotor subtype during follow-up. Further studies of consistency in PD subtyping methods should be conducted in the future.

## Introduction

Parkinson’s disease (PD) is a multisystem neurodegenerative disease that features predominant motor and nonmotor symptoms. Patients with PD exhibit huge individual differences in their motor and nonmotor characteristics. Previously, efforts have been made to distinguish the clinical subtypes of PD and the prognosis associated with the different subtypes (Hendricks and Khasawneh, 2021). In terms of motor symptoms, patients can be classified into tremor-dominant (TD), postural instability and gait difficulty (PIGD), and indeterminate subtypes (Stebbins et al., 2013). However, patients may transfer from one subtype to another as the disease progresses (Eisinger et al., 2020). By two years after baseline, around half of patients have experienced a change in their motor subtype (Erro et al., 2019). von Coelln et al. (2021) found that 50% of patients with the TD subtype and 60% of patients with the indeterminate subtype had shifted to the PIGD subtype by five years after baseline, indicating that most transfers are from a milder to a more severe subtype. However, inconsistencies in the motor subtypes, as observed via cross-sectional evaluation, have raised the question that these subtypes might simply reflect longitudinal disease progression (Simuni et al., 2016; Erro et al., 2019; Luo et al., 2019; von Coelln et al., 2021).

Recent evidence has shown that nonmotor PD symptoms are multidimensional, and that the prevalence and severity of these symptoms are diverse among patients (Ou et al., 2021). Classification methods based on both motor and nonmotor symptoms have been developed (Fereshtehnejad et al., 2015, 2017). In this context, patients can be categorized into mild-motor–predominant, intermediate, or diffuse malignant subtypes according to the severity of motor symptoms, the presence of rapid eye movement sleep behavior disorder (RBD), and the status of autonomic function and cognition (Fereshtehnejad et al., 2017). This kind of classification has been widely used in subtype studies (Fan et al., 2021; Mestre et al., 2021). Patients with the diffuse malignant subtype have been found to experience a faster deterioration of motor and cognitive symptoms and a shorter progression time to disease milestones (e.g., regular falls, dementia, wheelchair dependence, and placement in residential or nursing home care) and death (Fereshtehnejad et al., 2017; De Pablo-Fernandez et al., 2019). However, a recent study found that patients with advanced disease had a higher prevalence of the diffuse-malignant subtype compared to those who had shorter disease duration or lower disease staging (Erro et al., 2020). In addition, no significant differences were found between Lewy pathology staging and Alzheimer’s disease–related pathology staging among the different subtypes (De Pablo-Fernandez et al., 2019). Similar to the motor subtypes, the motor-nonmotor classification method has also made use of cross-sectional data, and the consistency of the subtypes in patients with early-stage PD still requires further investigation.

The previous review pointed out that, in order to support the hypothesis that the subtype accurately represents the underlying pathological characteristics, subtypes must exhibit consistency (Fereshtehnejad and Postuma, 2017). In the present study, we evaluate the consistency of the previously established subtypes in a large multicenter longitudinal cohort. We also explore the association between the subtype at baseline and the subtype at follow-up, as well as the potential determinants of change in subtype.

## Materials and methods

### Study design and patient inclusion

The Parkinson Progression Marker Initiative (PPMI) study was a prospective, multicenter observational cohort study whose cohort was used to develop the motor-nonmotor subtyping method. We therefore used the same cohort to evaluate the consistency of the motor-nonmotor subtypes (Fereshtehnejad et al., 2017). The details of the PPMI study design have been described elsewhere (Parkinson Progression Marker, 2011).

The inclusion criteria for the present study were: (i) patients diagnosed with PD and having dopaminergic deficit, as confirmed by dopamine transporter imaging; (ii) patients with a disease duration ≤ 3 years and Hoehn and Yahr stage < 3 at baseline. Patients with missing data at baseline in the scales used for the classification were excluded.

The PPMI study was approved by the institutional review boards at each participating PPMI site. Written informed consent was obtained from all participants in the study.

### Measurements

Patients were scheduled to be followed up for five years, so we used data from baseline as well as from all five years of follow-up. Face-to-face evaluations were conducted by experienced neurological doctors. Demographic features including age, age of onset, sex, and disease duration were recorded. Motor and nonmotor symptoms were evaluated by the following: the Movement Disorder Society-sponsored revision of the Unified Parkinson’s Disease Rating Scale (MDS-UPDRS); the Hoehn and Yahr scale; the Rapid Eye Movement Sleep Behavior Disorder Screening Questionnaire (RBDSQ); the Geriatric Depression Scale; the State-Trait Anxiety Inventory; the Questionnaire for Impulsive-Compulsive Disorders; the Scales for Outcomes in Parkinson’s Disease–Autonomic; and the Epworth Sleepiness Scale. Activities of daily living were assessed by the modified Schwab and England Activities of Daily Living scale. Cognitive evaluation was conducted using the following: the Montreal Cognitive Assessment; the 15-item version of Benton Judgment of Line Orientation (BJLO) test; the Hopkins Verbal Learning Test–Revised (HVLT-R); the Letter-Number Sequencing (LNS) task; a modified Semantic Fluency (SF) test; and the Symbol Digit Modalities Test (SDMT).

### Subtyping

Patients were classified into different subtypes at baseline, and the classification was repeated during follow-up, according to the motor and nonmotor symptoms. Because the motor-nonmotor subtyping method depends on the quartile level of disease severity of the whole group, as well as on cross-sectional data, we included all the patients available at baseline in the subtyping process. The subtyping process during follow-up also used all available patients, and those without baseline subtype information were excluded.

The method of motor-nonmotor subtype classification is described in the previous review (Fereshtehnejad et al., 2017). In brief, patients with motor scores >75th percentile and ≥1 of 3 nonmotor scores >75th percentile, or with all 3 nonmotor scores >75th percentile, were classified into the diffuse malignant subtype, and patients with motor and all three nonmotor scores <75th percentile were classified into the mild-motor–predominant subtype. Those who did not meet the aforementioned criteria were classified into the intermediate subtype. Motor scores (off-state) were calculated via the means of the Z-scores of the MDS-UPDRS part II, MDS-UPDRS part III, and PIGD score. The PIGD score was calculated as the mean of the scores of items 2.12, 2.13, 3.10, 3.11, and 3.12 of the MDS-UPDRS (Stebbins et al., 2013). The three nonmotor scores were the Z-scores of the RBDSQ, SCOPA-AUT, and cognitive tests. The cognition score was the mean of the Z-scores of the BJLO, HVLT-R (i.e., the mean score of the total recall, delayed recall, retention, and recognition discrimination index), SDMT, and combined scores (i.e., the mean score of the SF and LNS). In addition, we also divided patients into different motor subtypes according to the ratio of TD and PIGD scores (Stebbins et al., 2013). Patients with TD/PIGD score ratios ≥1.15 were classified as TD, those with ratios ≤0.90 were classified as PIGD, and the others were classified as indeterminate.

### Statistical analysis

Data were shown as median (quartile) for the continuous variables and as number (percent) for the categorized variables. The distribution was tested with Shapiro–Wilk tests. The Mann–Whitney U test and Chi-square test (or Fisher’s exact test) were used to compare the baseline characteristics of patients between groups, as appropriate, since the data were abnormally distributed. Cohen’s kappa (κ) and proportion of observed agreement were used to evaluate the agreement of the subtypes between baseline and follow-up. Cohen’s kappa value can be interpreted as following: κ < 0, no agreement; κ = 0–0.20, poor agreement; κ = 0.21–0.40, fair agreement; κ = 0.4–0.60, moderate agreement; κ = 0.61– 0.80, substantial agreement; and κ = 0.81–1, excellent agreement. Binary logistic regression was used to evaluate the determinants of the diffuse malignant subtype at follow-up.

Patients were divided into two groups according to whether they were in the diffuse malignant subtype at the fifth year of visit. Baseline motor-nonmotor subtype and characteristics that were not used for the classification were set as the dependent variables. Statistical significance was set as *p* < 0.05, and all tests were two-sided. Statistical Product and Service Solutions (SPSS) software, version 22.0, was used to conduct all the analyses.

## Results

**FIGURE 1:**
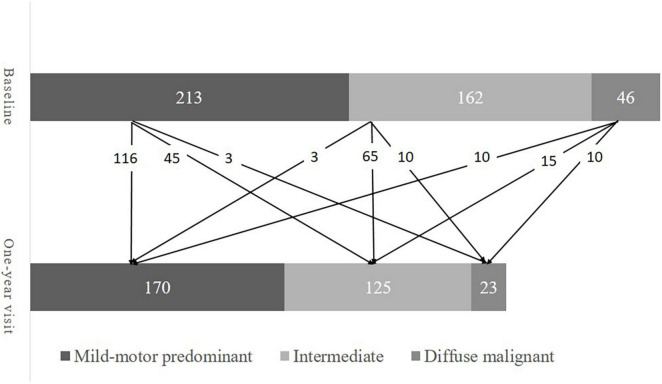
Subtype change from baseline to one-year visit.

A total of 421 patients were included in the present study. Their baseline demographics are presented in Table 1. About two-thirds (66.5%) of the patients were male. The median age at baseline and age of onset was 62.4 years and 61.2 years, respectively. At baseline, 213 patients (50.6%) were mild-motor–predominant, 162 patients (38.5%) were intermediate, and 46 patients (10.9%) were diffuse malignant. There were 318, 259, 241, 234, and 208 patients available for re-subtyping at each year’s follow-up, respectively. Subtype change between baseline and the one-year visit is displayed in Figure 1. At year five, 102 patients (49.0%) were classified as mild-motor–predominant, 88 patients (42.3%) were intermediate, and 18 patients (8.7%) were diffuse malignant. Only 120 patients (57.7%) had the same subtype at year five as at baseline. Specifically, 68 patients with mild-motor subtype at baseline (63.5%), 46 patients with intermediate subtype (52.9%), and 6 patients with diffuse malignant subtype (42.8%) had stable subtypes at year five. For the patients with mild-motor subtype at baseline, 33.6% and 2.8% of them, respectively, transferred to intermediate and diffuse malignant subtypes. For the intermediate patients, 36.8% and 10.3% of them, respectively, transferred to mild-motor–predominant and diffuse malignant. And 14.3% and 42.8% of diffuse malignant patients, respectively, transferred to the mild-motor–predominant and intermediate subtypes.

**TABLE 1:** Baseline characteristics of all patients.

	All patients (*n* = 421)	Mild–motor (*n* = 213)	Intermediate (*n* = 162)	Diffuse malignant (*n* = 46)	*P*-value
Age (median, quartile)	62.4 (55.2–69)	62.3 (54.8–69)	62.1 (54.8–68.9)	64.4 (59.2–70.2)	0.402
Age of onset (median, quartile)	61.2 (53.7–67.5)	61.2 (53.5–67.3)	60.6 (53.4–67.1)	62.4 (57.5–68.2)	0.548
Male (*n*, %)	280 (66.5%)	133 (62.4%)	114 (70.4%)	33 (70.7%)	0.199
Disease duration, years (median, quartile)	15 (10–24)	14 (9–22)	15.5 (9.8–24.3)	22 (11–28.5)	0.007*
MDS-UPDRS part I score (median, quartile)	5 (3–8)	4 (2–6)	5 (3–8)	9.5 (5.8–13)	<0.001*
MDS-UPDRS part II score (median, quartile)	5 (3–8)	3 (2–6)	5.5 (3–9)	11.5 (7.8–14)	<0.001*
MDS-UPDRS part III score (median, quartile)	19 (14–26)	17 (12–23)	20 (14–26)	31 (26–38)	<0.001*
Hoehn and Yahr stage (*n*, %)					<0.001*
1	182 (43.2%)	105 (49.3%)	75 (46.3%)	2 (4.3%)	
2	239 (56.8%)	108 (50.7%)	87 (53.7%)	44 (93.7%)	
SE-ADL score (median, quartile)	90 (90–100)	95 (90–100)	90 (90–100)	90 (83.8–90)	
RBDSQ score (median, quartile)	5 (3–7)	4 (3–5)	6 (4–9)	8 (5–10)	<0.001*
SCOPA-AUT score (median, quartile)	8 (5–12)	7 (5–9)	11 (6–16)	14 (11–18)	<0.001*
GDS score (median, quartile)	2 (1–3)	2 (0–3)	2 (1–3)	2 (1–5)	0.004*
STAI score (median, quartile)	63 (52–76)	61 (51–74.5)	62 (52–77)	73.5 (58.8–97)	<0.001*
QUIP score (median, quartile)	0 (0–0)	0 (0–0)	0 (0–1)	0 (0–0)	0.003*
ESS score (median, quartile)	5 (3–8)	5 (3–8)	5 (3–8)	7 (3–10)	0.091
MoCA score (median, quartile)	27 (25–29)	27 (25–29)	27.5 (25–29)	27 (25–29)	0.891
BJLO score (median, quartile)	13 (11–14)	13 (11–14)	13 (12–15)	13 (11–14)	0.073
HVLT score: Total recall (median, quartile)	24 (21–27)	24 (21–27)	25 (20–28)	24 (20.8–26.3)	0.154
HVLT score: Delayed recall (median, quartile)	8 (7–10)	8 (7–10)	9 (6.8–11)	8 (6–10)	0.230
HVLT score: Retention (median, quartile)	0.9 (0.7–1)	0.9 (0.7–1)	0.9 (0.7–1)	0.9 (0.7–1)	0.418
HVLT score: Discrimination recognition (median, quartile)	10 (9–11)	10 (9–11)	10 (9–11)	10 (8–11)	0.143
LNS score (median, quartile)	11 (9–12)	11 (8–12)	11 (9–13)	11 (10–12)	0.088
SF score (median, quartile)	47 (40–56)	47 (40–56)	48 (41–56)	47 (37.5–54)	0.614
SDMT score (median, quartile)	42 (34–48)	42 (34.5–47.5)	42.5 (34–48)	39.5 (31–45.3)	0.180

*Significant at 0.05.

MDS-UPDRS, Movement Disorder Society-sponsored revision of the Unified Parkinson’s Disease Rating Scale; RBDSQ: Rapid Eye Movement Sleep Behavior Disorder Screening Questionnaire; GDS: Geriatric Depression Scale; STAI: State-Trait Anxiety Inventory; QUIP: Questionnaire for Impulsive-Compulsive Disorders; SCOPA-AUT: Scales for Outcomes in Parkinson’s Disease–Autonomic; ESS: Epworth Sleepiness Scale; SE-ADL: modified Schwab and England Activities of Daily Living scale; MoCA: Montreal Cognitive Assessment; BJLO: Benton Judgment of Line Orientation, 15-item version; HVLT: Hopkins Verbal Learning Test–Revised; LNS: Letter-Number Sequencing task; SF: modified Semantic Fluency scale; SDMT: Symbol Digit Modalities Test.

The kappa value indicated a fair agreement between the baseline and follow-up subtypes (Table 2). The kappa values of the baseline and follow-up motor subtypes are also listed for comparison. With disease progression, the kappa value of the motor-nonmotor subtype showed a mild decrease, but the kappa value of the motor subtype fell from a moderate agreement to a fair agreement. In addition, the total agreement of the motor-nonmotor subtype was lower than that of the motor subtype during follow-up.

**TABLE 2:** Consistency of subtypes during follow-up compared to baseline.

	Number	Motor-nonmotor subtype		Motor subtype	
			
		Kappa value ± SD	Total agreement	Kappa value ± SD	Total agreement
1-year visit	318	0.30 ± 0.09	192 (60.6%)	0.46 ± 0.09	229 (72.0%)
2-year visit	259	0.26 ± 0.10	148 (57.1%)	0.40 ± 0.10	181 (69.9%)
3-year visit	241	0.24 ± 0.11	136 (56.4%)	0.32 ± 0.10	160 (66.4%)
4-year visit	234	0.29 ± 0.11	139 (59.4%)	0.23 ± 0.10	143 (61.1%)
5-year visit	208	0.25 ± 0.12	120 (57.7%)	0.23 ± 0.10	122 (58.6%)

Patients completing their fifth-year visit were younger and had lower ages of onset, lower MDS-UPDRS part II and III scores, and greater BJLO and SDMT scores compared to those without a fifth-year visit. To minimize the influence of the loss of follow-up, we performed a sensitivity analysis that included only the 208 patients who completed a fifth-year visit. The 208 patients were reclassified into a new motor-nonmotor subtypes at baseline and during the follow-up based on the data of these 208 patients. The agreements between the old and new motor-nonmotor subtypes were compared and the kappa agreement indicated a substantial to excellent agreement (data not shown). The results of the agreement between the new baseline and follow-up subtypes were also similar to the previous results (Supplementary Table 1).

Logistic regression analysis showed that only baseline subtypes were the determinants of having the diffuse malignant subtype by the fifth-year visit. Having the diffuse malignant (OR = 26.00; 95% CI, 5.46–123.90; *p* < 0.001) or intermediate (OR = 4.00; 95% CI, 1.05–15.26; *p* = 0.042) subtype at baseline was associated with a higher risk of being classified as diffuse malignant by the fifth-year visit, compared to mild-motor–predominant patients.

## Discussion

In the present study, we evaluated the agreement of clinical subtypes from baseline through five years of follow-up in a large multicenter cohort with early-stage PD. A fair degree of agreement was observed, and only 120 patients (57.7%) had the same subtype at baseline and at the fifth-year visit. The mild-motor–predominant subtype had the highest proportion of stable patients, followed by the intermediate and diffuse malignant subtypes. Although the motor-nonmotor subtype was inconsistent, having the intermediate or diffuse malignant subtype at baseline was related to an increased risk of having the diffuse malignant subtype five years later.

The total agreement of motor-nonmotor subtype between baseline and follow-up (57.7%) was higher than the agreement of motor-nonmotor subtypes in a cohort with late-stage PD (35.3%) (Ygland Rodstrom and Puschmann, 2021). This study also had longer intervals between the baseline and re-subtyping, and only 38% of baseline patients were included in the re-subtyping (Ygland Rodstrom and Puschmann, 2021).

In the present study, the overall agreement of the motor subtypes decreased with disease progression, which is consistent with previous studies of motor subtypes (Kohat et al., 2021). Shifts in both directions (i.e., from less to more severe, and vice versa) were observed, and shifts in subtype were influenced by the degree of disease progression during follow-up. Those with faster deterioration tended to transfer from the mild subtype to the severe subtype, and those with lesser deterioration tended to transfer from the severe subtype to the mild subtype. Such bidirectional transfer has also been observed among the motor subtypes (Erro et al., 2019; Lee et al., 2019; Luo et al., 2019). Although patients tended to fall into the PIGD type in the advanced disease stage in the previous studies, some patients with the PIGD subtype at baseline were re-classified into the TD or indeterminate subtypes during follow-up because of increased tremor scores (Erro et al., 2019; von Coelln et al., 2021). In addition, compared to the motor subtype, the motor-nonmotor subtype showed a lower degree of consistency, even in the first year of follow-up, and had lower total agreements with baseline through the five years of follow-up.

In developing the subtyping method, one of the most important clinical considerations for us was that it could predict individual prognosis in the early stages of the disease. The prognostic value of the current motor-nonmotor subtype has been proven in patients with early- and late-stage PD (Fereshtehnejad et al., 2017; De Pablo-Fernandez et al., 2019; Ygland Rodstrom and Puschmann, 2021). Patients in these studies with the intermediate or diffuse malignant subtype had a shorter progression time to dementia and death compared to mild-motor–predominant patients (De Pablo-Fernandez et al., 2019). These patients also had varying disease duration. The results of the present study indicate that there is a need to re-subtype and update the prognosis after even one year from baseline. In addition, the results of subtyping studies should be compared carefully, as they used different inclusion criteria and the characteristics of patients classified in each subtype varied between studies.

Another important aim of subtyping is to identify distinct disease entities (Fereshtehnejad and Postuma, 2017). In this regard, consistency in subtyping can be an important indicator of disease entities, but it has rarely been examined in subtyping studies (Hendricks and Khasawneh, 2021). Our results revealed that motor-nonmotor subtypes change from one to another during follow-up. Future studies using predefined methods or data-driven methods for clustering should confirm the consistency of these subtypes.

Internal and external limitations can explain the inconsistencies of the motor-nonmotor subtype. First, the definition of the motor-nonmotor subtype did not include the effects of treatment on the nonmotor symptoms. We used off-state data to eliminate the effects of dopamine replacement therapy on patients’ motor symptoms. However, several nonmotor symptoms such as rapid eye movement sleep behavior disorder (RBD) and constipation can also be improved by drugs or non-medicinal treatments (Grimes et al., 2019). Treatment can decrease the severity reported by patients and decrease the differences between patients.

Moreover, patients who completed the fifth-year visit had lower MDS-UPDRS part II and part III scores compared to those without the fifth-year visit, which added to the inconsistency of the subtypes between baseline and follow-up. To test the influence of the loss of follow-up on baseline–follow-up consistency, we performed a sensitivity analysis that included only patients with a fifth-year visit. The result of this analysis showed similar results, i.e., that the consistency between the baseline and follow-up subtypes was not good. This indicated that the loss of follow-up was not the reason for the inconsistency.

Another important limitation was that the current motor-nonmotor subtyping criteria was based on an individual’s position within the group. Individual subtype at follow-up was determined not only by individual progression, but also by the speed of deterioration of other patients in the group. This added difficulty in interpreting the shifts between the subtypes and reduced the applicability of motor-nonmotor subtyping methods in the follow-up. This limitation has also been found in other subtyping criteria using data-driven methods and cross-sectional data (Erro et al., 2016; Lawton et al., 2018; Zhang et al., 2019). Methods based on absolute values or ratios (such as the motor subtypes) would not be influenced by the progression of others in the group and are therefore more suitable for analyzing the consistency of subtypes in follow-up (Stebbins et al., 2013). We plan to develop a new subtyping method based on the absolute values or ratios of motor and nonmotor symptom scale scores and establish its applicability in the longitudinal cohort in future studies.

Our study also had several strengths. We used data from the same cohort that was used to develop the motor-nonmotor subtyping criteria. This is a high-quality longitudinal cohort, and its use therefore has the potential to increase the reliability of our results. We also adopted comprehensive assessments for the classification, which increased the accuracy of subtyping (Ygland Rodstrom and Puschmann, 2021).

In conclusion, we found that motor-nonmotor subtypes were not fixed, but rather changed during follow-up in patients with early-stage PD, and that the agreement of the motor-nonmotor subtypes was lower than that of the motor subtypes. The inconsistency of motor-nonmotor subtypes suggests that they are not distinct disease entities. Future clustering studies should devote more attention to the consistency of subtypes.

## Data availability statement

The datasets presented in this study can be found in online repositories. The names of the repository/repositories and accession number(s) can be found below: All the data was available from the PPMI database with the consent of the PPMI Data and Publications Committee.

## Ethics statement

The PPMI study was approved by the institutional review boards at each participating site. The patients/participants provided their written informed consent to participate in this study.

## Author contributions

YX: statistical analysis, design, execution, review and critique, manuscript writing of the first draft, and review. QW, RO, YH, LZ, KL, JL, TY, and QJ: manuscript review. HS: statistical analysis review and critique and manuscript review. All authors contributed to the article and approved the submitted version.
