# Supplementary material for: Stability of motor-nonmotor subtype in early-stage Parkinson’s disease
Source: Front Aging Neurosci. 2022 Nov 10;14:1040405. doi: 10.3389/fnagi.2022.1040405 (PMC9686273; doi:10.3389/fnagi.2022.1040405)
Supplement: Supplementary file 1 [file Table_1.DOCX]

**Supplementary table 1 sensitive analysis of the consistency of subtypes during follow-up compared to baseline.**

|  | Number | Motor-nonmotor subtype |  |
| --- | --- | --- | --- |
|  |  | kappa value ± SD | total agreement |
| 1-Year visit | 178 | 0.36±0.12 | 111(62.4%) |
| 2-Year visit | 171 | 0.23±0.12 | 93(54.4%) |
| 3-Year visit | 179 | 0.22±0.12 | 97(54.2%) |
| 4-Year visit | 186 | 0.28±0.12 | 108(58.1%) |
| 5-Year visit | 208 | 0.25±0.11 | 117(56.3%) |
